# Formulation and production of persimmon milk drink and evaluation of its physicochemical, rheological, and sensorial properties

**DOI:** 10.1002/fsn3.2772

**Published:** 2022-02-11

**Authors:** Akbar Jokar, Mohamad Hossyn Azizi

**Affiliations:** ^1^ Food Science, Agriculture Engineering Research Department Fars Agriculture Research Center Shiraz Iran; ^2^ Department of Food Science College of Agriculture Tarbiat Modares University Tehran Iran

**Keywords:** drink, formulation, persimmon milk, physicochemical, rheology, sensorial properties

## Abstract

One of the effective ways for increasing milk intake in a diet is producing flavored milk. In the present study, flavored persimmon milk drinks were formulated and produced. Rheological, sensorial, and physicochemical properties of the drinks were evaluated. Different amounts of persimmon (5, 10, 20, and 30% W/V), gum Arabic (0.1 and 0.2% W/V), and sugar (3 and 5% W/V) were used to produce persimmon milk. All the experiments were done in a completely randomized design (CRD) in three replicates. First, persimmons were mixed thoroughly in a blender then, according to the formulation, whole milk was added to it and mixed again. The obtained mixture was heated up to 50 ^ᵒ^C then sugar and the gum were added and mixed completely for 3 min. The drink was pasteurized at 90 ^ᵒ^C for 1 min. Sensorial analysis revealed that the most acceptable persimmon milk was related to 0.1% gum Arabic, 5% sugar, and 10% persimmon. Flow behavior of the drinks with 20 and 30% persimmon was Non‐Newtonian, while 10% persimmon samples with and without gum showed Newtonian behavior. The drinks with 20 and 30% persimmon were pseudoplastic, and their apparent viscosity increased by increasing shear rates. By applying a proper content of persimmon in milk, we can produce a nutritious flavored milk drink with acceptable taste, stability, and consistency. As this drink has high nutrient contents like phenol, dietary fiber, vitamins, antioxidants, etc., it can help promote health, especially for children.

## Graphical abstract

Different amounts of persimmon (5, 10, 20, and 30% W/V), gum Arabic (0.1 and 0.2% W/V), and sugar (3 and 5% W/V) were used to produce persimmon milk. By applying a proper content of persimmon in milk, we can produce a nutritious flavored milk drink with acceptable taste, stability, and consistency. As this drink has high nutrient contents like phenol, dietary fiber, vitamins, antioxidants etc., it can help promote health, especially of children.

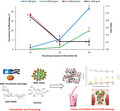

## 1 INTRODUCTION

Milk is a major part of various flavored milks. Nutrient ingredients and outstanding nutritional effects of milk have been always valuable for human beings. Humans had early found that both children and adults must consume this rich food. Milk primarily consists of water, protein, fat, lactose, minerals, and some amounts of vitamins. Therefore, it provides all the necessary nutrients for the growth and nourishment of people. Flavored milk beverages being a core food in the Australian Dietary Guidelines can make a significant contribution to an adult healthy diet because of their essential nutrients, vitamin D, and crucial minerals (especially calcium). It should be noted that low consumption of milk and dairy products can cause serious deficiency of these nutrients (Ha et al., 2009; Hong et al., 2013; Patel et al., 2018; Rosemont et al., 2000; Yin & Wang, 2007). One of the effective ways to increase milk intake in a diet is producing flavored milks. Nutritional features of milk and fruits can be relatively preserved in flavored milks. Researchers maintain that flavored and fruit milks have higher nutritional value in comparison with ordinary milk and nonflavored fruit juices (Laparra et al., 2008; Mann et al., 2014). There are many studies about the high bioavailability of bioactive and minerals compounds in fruit‐flavored milk drinks in literature. They announced that adding fruits to milk especially with high fat, significantly enhanced Zinc and Iron, carotenoids, and other photochemical bioavailability, their transportation and uptake in vitro, compared to ordinary fruit juice/milk. Furthermore, flavored milk drinks can alleviate low consumption of ordinary milk, especially in children (Cilla et al., 2012; Garcia‐Cayuela et al., 2018; García‐Nebot et al., 2009, 2010; García‐Nebot et al., 2010; Jouki et al., 2021; Lemmens et al., 2014; Mahato et al., 2021; Oduro et al., 2021). The development of new flavored milk beverage formulations which are highly accepted by consumers is one of the driving forces of dairy factories. Many kinds of fruit‐flavored milk beverages exist in the market, but new formulations with highly nutritious fruits are also required to compensate nutritional deficiency and also small market proportion.

Persimmon (*Diospyros kaki* L.) is a widespread, sweet, and highly nutritional fruit containing high quantities of carotenoids, besides minerals (Mg and P), vitamins (A, B, and C), dietary fibers and pectic substances, condensed tannins, and high sugar content (Altuntas et al., 2011; Hernández‐Carrión et al., 2014; Lee et al., 2010). Persimmon is one of the most bio‐active nutritious fruits to a large extent due to its dietary fiber and phenolic compounds. Bioactive compounds especially phenolics (ferulic, *p*‐coumaric, and gallic acids) and carotenoids (*β*‐cryptoxanthin, lycopene, *β*‐carotene, and lutein) are of major interest in persimmon fruit. The strong antioxidant potential of these bioactive compounds can play a significant role in preventing and curing various diseases like diabetes, hypercholesterolemia, and cancer (Cortellino et al., 2009; Karaman et al., 2014; Yaqub et al., 2016). The shelf life of the matured persimmon is quite short (nearly around 4 weeks). To extend persimmon availability, it could be processed into new and charming products (dairy‐flavored beverages, smoothies, or fruit desserts) which demonstrate a potential way to contribute to consumers’ health in terms of carotenoids and other bioactive intakes. In this way, improving the nutritional, physicochemical, and textural properties of flavored milks is possible (Cortellino et al., 2009; Garcia‐Cayuela et al., 2018; Hernández‐Carrión et al., 2014).

There are lots of reports in the literature about the development of new fruit‐flavored milk but nearly no one is about persimmon milk beverage, especially the optimum amounts of ingredients, formulation, stability, etc. Cortellino et al., (2009) used persimmon puree as a thickening and creaming agent in sherbet. Persimmon puree was a suitable thickening and creaming agent for sherbet with high nutritional quality due to the high polyphenol content in persimmon (Cortellino et al., 2009). Kamaran et al., (2014) applied persimmon puree into ice cream. They reported that better properties and textural characteristics were related to the samples containing persimmon. Bioactive properties of the samples with persimmon were drastically improved (Karaman et al., 2014). Hernández‐Carrion et al., (2014) incorporated freeze‐dried persimmon into milk for producing flavored milk. The new fruit milk beverages were prepared and treated with high‐pressure technology. These samples showed the best rheological properties as they did not form a gel structure or precipitate down (Hernández‐Carrión et al., 2014). Physicochemical, sensory, and microbial properties of new formulated strawberry‐flavored milks were evaluated during 14 days of storage at the refrigerator. Physicochemical properties and sensorial acceptance of strawberry‐flavored milk declined with the increase in time (Hossin et al., 2021). Jalilzadeh‐Afshari and Fadaei, (2021) reported that increasing the Gaz‐angubin content and bitter orange peel extract in flavored milk improved sensorial properties, viscosity, antioxidant activity, and total polyphenol content, while decreased total microbial count. The best flavored milk sample contained 15% Gaz‐angubin and 0.075% bitter orange peel extract (Jalilzadeh‐Afshari & Fadaei, 2021). Patel et al., (2020) formulated value‐added flavored buffalo milk and pumpkin pulp. The sterilized flavored pumpkin buffalo milk showed a decline in color and appearance scores during storage at room temperature after day 90. The nutritional qualities were not affected highly during the 180‐day storage period (Patel et al., 2020).

This study was first designed to produce and formulate persimmon‐flavored milk, and then to investigate the effects of persimmon content, sugar, and gum on physicochemical, sensorial, and rheological properties of the new flavored milk beverage.

## 2 MATERIALS AND METHODS

### 2.1 Materials

Fresh whole milk was purchased from a local selling milk center in Tehran and well‐ripened persimmon, Hachiya Variety, was prepared from gardens in Talkhedash, Shiraz, Iran. Sugar was purchased from a local store. Gum Arabic and all the other chemicals were provided by Merck Company.

### 2.2 Preparing and processing of persimmon milk drink

In order to optimize formulation, sample preparation was conducted in two steps. In both steps, dairy beverages were prepared as follows: persimmon, gum Arabic, and sugar weighed according to the formulation for preparing 1 L of the dairy drink. Persimmons were blended and homogenized thoroughly in a usual blender for 1 min (Blender Nautiunl MJ‐176 NR). Then, 500 ml of the milk was added and mixed with the persimmon completely for 1 min. The remaining milk was added and mixed again for 1 min. The obtained mixture was heated up to 50 ^ᵒ^C, gum and sugar were added and mixed to the drink for 3 min. Finally, the obtained drink was pasteurized at 90 ^ᵒ^C for 1 min and cooled quickly up to ambient temperature (Branger et al., 1999; Demott, 1975; Jokar et al., 2006).

First, samples were prepared with whole milk and different amounts of persimmon (10, 20, and 30% W/V), gum Arabic (0.2% W/V), and sugar (3% W/V). In the second step, considering the results of the first sensory evaluation, the amounts of sugar and gum Arabic were selected as 5 and 0.1%, respectively. Finally, four samples with 5, 10, 20, and 30% persimmon were prepared with 5% sugar and 0.1% gum Arabic contents.

### 2.3 Sensory properties

In the first sensorial evaluation of three samples, the taste panel was asked to assess the taste, and consistency of the samples at 7^ᵒ^C. The results showed that the taste scores were low, and the consistencies of the samples were so high and unpleasant, so we concluded that the samples did not have acceptable taste and consistency. Undesirable mouth feeling was reported, especially for 20 and 30% persimmon samples. Therefore, in the second sensory evaluation, samples were produced with 5% sugar and 0.1% gum Arabic (Demott, 1975; Jokar et al., 2006).

Samples from the second step were evaluated in terms of taste, color, consistency, and overall acceptability. Sensorial evaluations were performed by 14 trained test panels with 5‐point Hedonic Scaling Test: 1 for dislike extremely, 2 for dislike, 3 for like or dislike, 4 for like, and 5 for like extremely (Branger et al., 1999; Jokar et al., 2006; Maynard et al., 1965; Watts et al., 1989).

### 2.4 Physicochemical properties

Color of the drinks and whole milk (*Lab* factors) measured in a portable sphere spectrophotometer set (Lovibond‐SP60, England), previously calibrated by the white and black standard. The test was performed in duplicate with samples of 30 ml. The trend of color change (*Lab* factors) due to the addition of persimmon to whole milk was evaluated.

In order to appraise the stability of the samples, after cooling the drinks to 15 ^ᵒ^C, 100 ml of them was poured in a 100‐mL lab cylinder. The cylinders were put in the refrigerator (5^ᵒ^C), and the amount of precipitation of the drinks was measured daily for a week (Demott, 1975; Jokar et al., 2006).

Dry solids and protein of whole milk, persimmon, and the drinks were measured by AOAC (16.032) and AOAC (16.036), respectively (Horwitz, 1980). Fat of the whole milk was determined by Gerber method (Horwitz, 1980; Jelen et al., 1987). Total soluble solids (TSS) of persimmon were determined by a digital refractometer (Kyoto Company, Kyoto, Japan).

### 2.5 Rheological properties

Drink samples with 10, 20, and 30% persimmon contents with and without gum were produced, according to the mentioned procedure. Flow measurements were performed in an MYR viscometer (MYR, V2R Spanish) by applying an R2 spindle (diameter of 4.7 centimeters). Three days after keeping samples in a refrigerator (5^ᵒ^C), 400 ml of the drinks was poured in a 500‐mL beaker, and as the viscometer had only three shear rates (60, 100, and 200 rpm), their apparent viscosity was measured in the defined shear rates. Shear stress (Ʈ) was calculated at the correspondent shear rate (G), and initial shear stress or yield stress (Ʈ_0_) was determined by Casson model (Ʈ^1/2^_=_Ʈ_0_^1/2^_+_CG^1/2^, where C is constant). Measurements were done in triplicate at room temperature (25^ᵒ^C).

The obtained data were fitted to the Herschel–Bulkley model (Ʈ_=_Ʈ_0+_K (G)^m^). A linear correlation of log Ʈ_‐_Ʈ_0_ versus. log G was used for getting consistency coefficient (K) and flow index (m). The effect of gum on K and m was also evaluated (Barnes, 2000; Bourne, 2002; Keshtkaran et al., 2013; Ramírez‐Sucre & Vélez‐Ruiz, 2011).

### 2.6 Statistical analysis

All the experiments were done in a completely randomized design in three replicates, and data were analyzed with SPSS20 software. Flow indexes and consistency coefficients in the drinks with and without gum were compared using the Student's *t*‐test.

## 3 RESULTS

Important and major components of milk, persimmon, and persimmon milk drinks are presented in Tables 1 and 2.

**TABLE 1:** Comparison of milk and persimmon ingredients

	Protein (%)	Fat (%)	Dry solids (%)	pH	TSS
Persimmon	2.2	N.D	32	5.34	29
Milk	3.4	3.43	12.2	6.78	N.D

Abbreviations: Nd, Not Determined; TSS, Total Soluble Solids.

**TABLE 2:** Ingredients of persimmon milk drinks

	Persimmon	Whole milk	5% persimmon	10% persimmon	20% persimmon	30% persimmon
TSS (%)	29	12.2	17.98	18.68	19.93	20.98
Protein (%)	2.2	3.4	3.4	3.27	3.1	2.9

### 3.1 Sensorial evaluations

The nonparametric analyses of the results from the `p (3% sugar and 0.2% gum Arabic) are shown in Table 3. As it can be seen in Table 4, taste and consistency were not significantly different (*p* > .05). The taste panel announced that the taste and consistency of all the drinks were unpleasant.

**TABLE 3:** Nonparametric analysis results from the first‐step sensory evaluation

Evaluated features	Treatments (Persimmon)	Number	Mean ranks
Taste	10%	14	17.69
	20%	14	26.29
	30%	14	20.25
	Total	49	
Consistency	10%	14	24.57
	20%	14	21.04
	30%	14	18.89
	Total	49	

**TABLE 4:** Nonparametric analysis results from the second‐step sensory evaluation

Evaluated features	Treatments (Persimmon)	Numbers	Mean ranks
Taste	5%	14	19.57
	10%	14	40.14
	20%	14	27.86
	30%	14	26.43
	Total	56	
Color	5%	14	23.43
	10%	14	34.39
	20%	14	26.29
	30%	14	29.89
	Total	56	
Consistency	5%	14	26.86
	10%	14	39.75
	20%	14	26.68
	30%	14	20.71
	Total	56	

**FIGURE 1:**
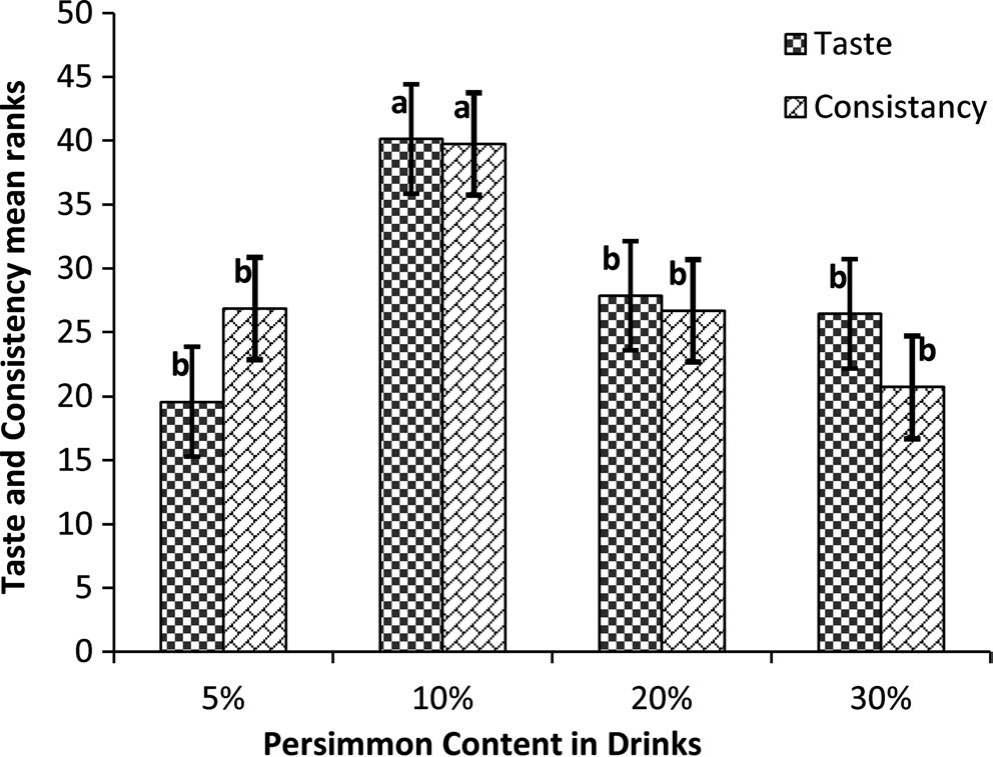
Mean comparison of taste and consistency scores

The results of the second‐step sensorial analyses are shown in Table 4. Mean comparison results are shown in Figure 1.

### 3.2 Color

**FIGURE 2:**
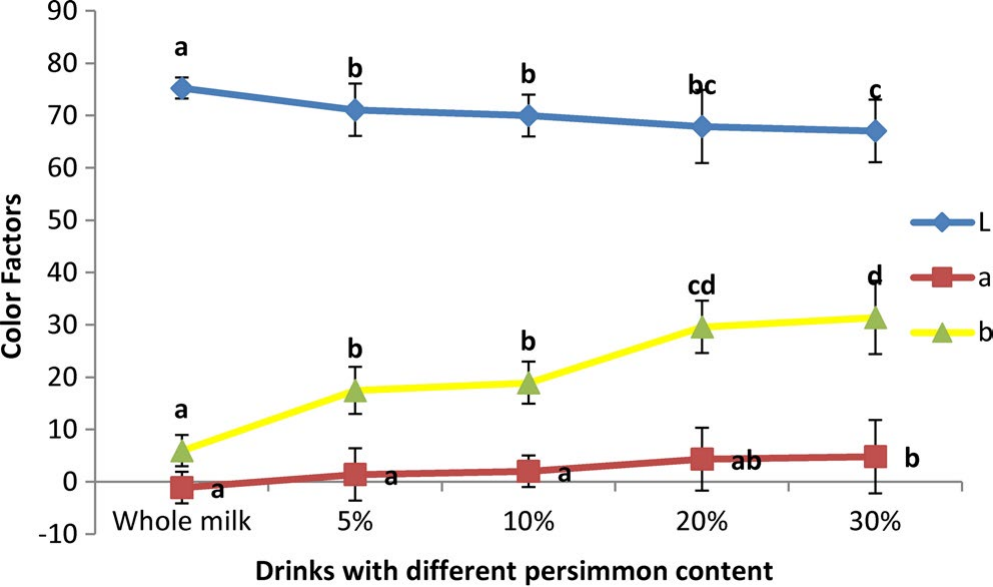
Color change trends in the drinks

Color change trends, due to the addition of persimmon to whole milk, are shown in Figure 2.

### 3.3 Drink stability

**FIGURE 3:**
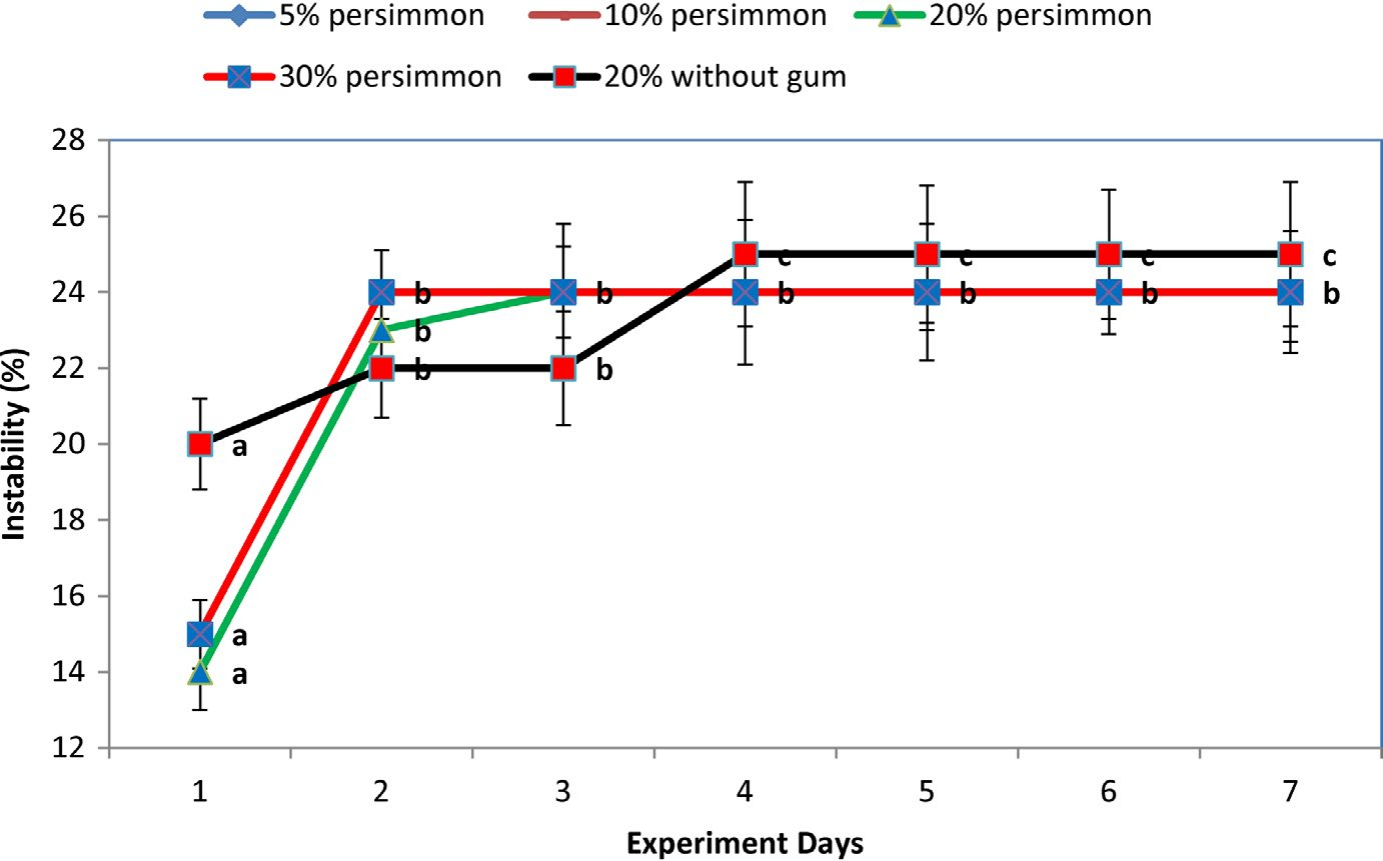
Stability of the drinks in different days of experiments

The stability of different samples on different days was not similar. Figure 3 shows these results. Samples with 5 and 10% persimmon contents were completely stable, while 20 and 30% samples indicated instabilities.

### 3.4 Rheological properties

Fluid behaviors of samples with 20 and 30% persimmon contents with and without gum were Non‐Newtonian, while the drinks with 10% persimmon content with and without gum had Newtonian behavior. Tables 5 and 6 show apparent viscosities in different shear rates.

**TABLE 5:** Apparent viscosity of the drinks with the gum at different shear rates

Shear Rates (1/S)	Samples (Persimmon Content)	Apparent viscosity (Pa.S)
21.3	10%	0.079 ± 0.0003^a^
	20%	1.605 ± 0.013^b^
	30%	4.087 ± 0.031^c^
35.6	10%	0.065 ± 0.001^a^
	20%	1.198 ± 0.013^b^
	30%	2.960 ± 0.020^c^
71.1	10%	0.064 ± 0.0006^a^
	20%	0.727 ± 0.008^b^
	30%	2.140 ± 0.020^c^

Note

Different letters show significant differences (*p*<.05) in Duncan's comparison test.

**TABLE 6:** Apparent viscosity of the drinks without the gum at different shear rates

Shear Rates (1/S)	Samples (Persimmon Content)	Apparent viscosity (Pa.S)
21.3	10%	0.019 ± 0.0015^a^
	20%	0.161 ± 0.0010^b^
	30%	1.06 ± 0.020^c^
35.6	10%	0.018 ± 0.001^a^
	20%	0.119 ± 0.0006^b^
	30%	0.860 ± 0.20^c^
71.1	10%	0.021 ± 0.0006^a^
	20%	0.064 ± 0.0008^b^
	30%	0.096 ± 0.020^c^

Note

Different letters show significant differences (*p*<.05) in Duncan's comparison test.

**FIGURE 4:**
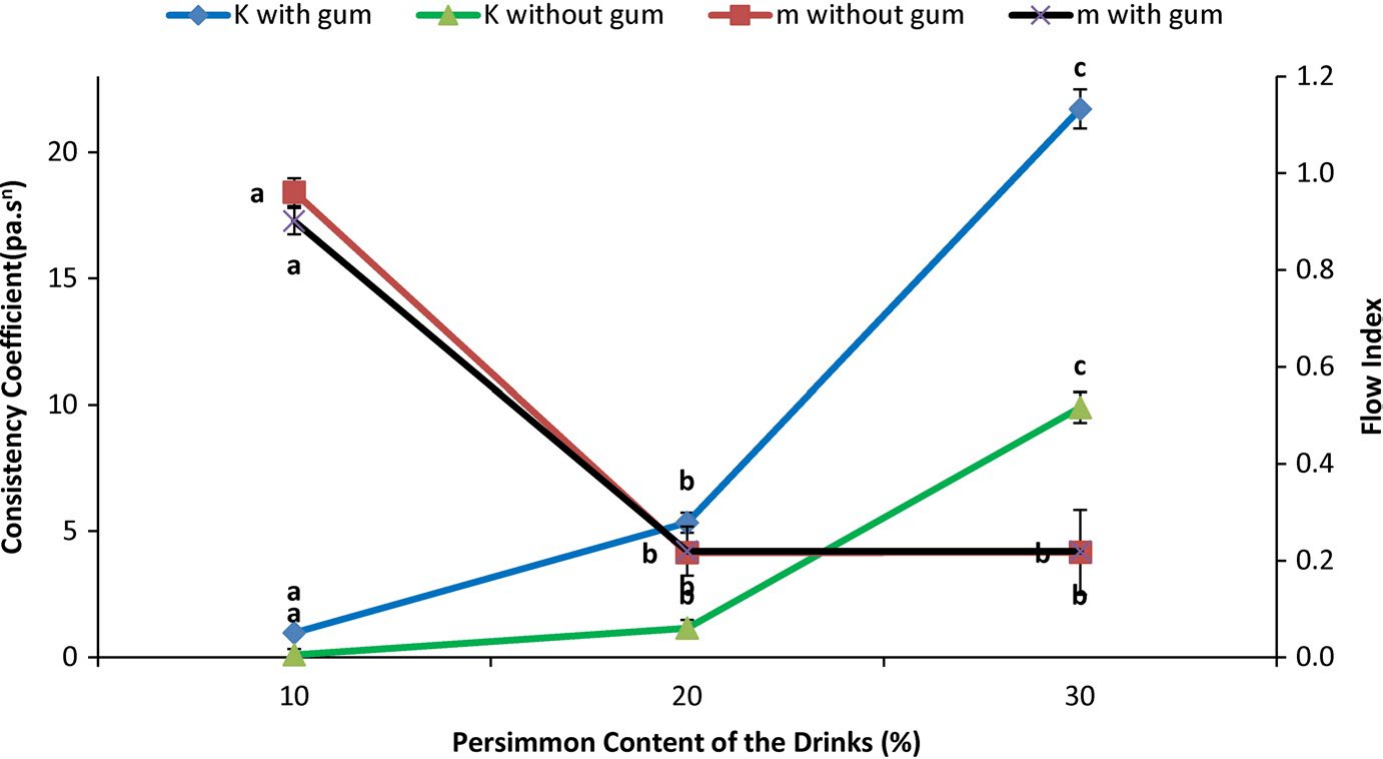
Effects of persimmon content and gum on consistency coefficient and flow index

Statistical analysis showed that persimmon content had a significant effect on yield stress, flow index, and consistency coefficient (Table 8) in all the drinks with and without gum (*p* < .05). Tables 7 and 8 show these results and analyses. Gum had a significant effect on K and m in all the drinks (*p* < .05). The effects of persimmon content on K and m are shown in Figure 4.

**TABLE 7:** The effects of persimmon contents and gum on flow index, consistency yield stress of the drinks in Herschel–Bulkley model

	Samples (Persimmon Content)	Flow Index (m)	K (pa.s^n^)	Yield stress (Pa)	R^2^
Without Gum	10%	0.961^a^ ± 0.019	0.098^a^ ± 0.012	0	0.894 ± 0.009
	20%	0.216^b^ ± 0.010	1.152^b^ ± 0.022	1.43 ± 0.0.044	0.97 ± 0.005
	30%	0.218^b^ ± 0.009	9.894^c^ ± 0.406	7.12 ± 0.617	0.986 ± 0.015
With Gum	10%	0.901^a^ ± 0.002	0.965^a^ ± 0.009	0	0.962 ± 0.005
	20%	0.219^b^ ± 0.009	5.332^b^ ± 0.199	25.357 ± 0.11	0.980 ± 0.012
	30%	0.218^b^ ± 0.005	21.72^c^ ± 0.775	114.906 ± 34.7	0.985 ± 0.003

Note

Different letters show significant differences (*p*<.05) in Duncan's comparison test.

*R* ^2^: coefficient of determination in Herschel–Bulkley model.

**TABLE 8:** Student's *t*‐test for evaluating flow index and consistency coefficient means equality test between drinks with and without gum

*t*‐test for Equality of Means, Equal variances assumed				
		t	*df*	Sig. (2‐tailed)
10% persimmon	K	−98.511	4	0.000
	m	28.326	4	0.000

*t*‐test for Equality of Means, Equal variances not assumed				
		t	*df*	Sig. (2‐tailed)
20% persimmon	K	−36.125	2.05	0.001
	m	−7.112	2.164	0.015

*t*‐test for Equality of Means, Equal variances assumed				
		t	*df*	Sig. (2‐tailed)
30% persimmon	K	−17.193	4	0.000
	m	−0.263	4	0.806

## 4 DISCUSSION

pH of persimmon (Table 1) was nearly the same as the results from Altuntas et al., (2011). TSS, dry solids, and protein content were dramatically higher than the reported data from Yaqub et al., (2016) and Cortellino et al. (2009). These differences are mostly due to the different varieties and regions of growth (Altuntas et al., 2011; Cortellino et al., 2009; Yaqub et al., 2016). As it can be seen in Table 2, TSS increased (from 12.2% in milk to 20.98%) by increasing persimmon content from 5% to 30%, while protein content declined. Increasing TSS is significant and might increase the consistency of the drinks, but can cause instability. Decreasing protein is not significant and worrying.

### 4.1 Sensorial properties

In the first sensorial analysis, the test panel announced that all the drinks were with low and unacceptable taste and consistency. There were no significant differences between samples (*p* > .05). Therefore, in the second sensorial test, sugar content increased from 2% to 5% and gum Arabic decreased from 0.2% to 0.1% in the drinks. As persimmon has dietary fiber and hydrocolloid substances, the drinks needed lower amounts of gum.

In the second sensorial analysis, there were not any significant differences among the colors of the drinks (*p* > .05), while the taste and consistency of the drinks were significantly different (*p* < .01). The best drink, in terms of taste and consistency, was found to be the drink with 10% persimmon, 5% sugar, and 0.1% gum Arabic. The mean comparisons of taste and consistency scores are shown in Figure 1. As shown in Figure 1, the 10% persimmon sample got the highest taste and consistency scores and there were significant differences among the samples (*p* < .05). Taste, color, and consistency scores increased by increasing persimmon content from 5% to 10%, while these sensorial scores declined with 10 and 20% persimmon content. These trends might be due to the new astringency and high creamy mouthfeel in the flavored milks with high persimmon content. The same sensorial trend and results were reported by Karaman et al., (2014), in the optimization of persimmon content in ice cream (Karaman et al., 2014).

### 4.2 Color

Persimmon changed the color of whole milk. Figure 2 shows the trend of color change in the drink. By increasing persimmon content, lightness (*L*) decreased while redness (*a*) and yellowness (*b*) increased. These changes in the color of the samples are due to the yellow pigments especially carotenoids from persimmon. The same trends in changing the color of persimmon‐flavored ice cream and pumpkin‐flavored milk were reported (Karaman et al., 2014; Patel et al., 2020).

### 4.3 Stability

As it can be seen in Figure 3, samples with 5 and 10% persimmon were so stable, and their stability remained constant until the end of the seventh day of storage. However, samples with 20 and 30% persimmon contents showed the highest instability on the second day (24%) and after that remained constant. The stability of samples without gum was similar to the corresponding sample with gum (like 20% in Figure 3). It shows that persimmon hydrocolloid substances had a positive effect on drink stability. The positive effects of dietary fibers, especially pectin of persimmon on the stability and texture of ice cream and sherbet were reported (Cortellino et al., 2009; Karaman et al., 2014).

Although pectin and other hydrocolloids increased by rising persimmon content, in comparison with the solid contents, it was not high enough to produce a firm jelly network for entrapping and keeping the solids stable. Therefore, it was expected that the higher persimmon content could result in more aggregation of solid particles and instability of the drinks in high persimmon content. The same results were reported in beverages containing low levels of pectin and high solid contents (Lucey et al., 1999; Sedlmeyer et al., 2004). Given the strong homogenization in dairy plants, gum and other stabilizers might be eliminated in producing such a flavored milk drink.

### 4.4 Rheological properties

Data analysis showed that by increasing shear rate, apparent viscosity decreased in all the samples with 20 and 30% persimmon contents (with and without gum). However, viscosity in the drinks with 10% persimmon content with and without gum remained nearly constant in different shear rates, indicating Newtonian behavior. The drinks with 20 and 30% persimmon contents did not follow Newtonian law and had yielded stresses. These drinks showed pseudoplastic behavior (Tables 5 and 6). Apparent viscosities in different shear rates and persimmon contents were significantly different (*p* < .05). Soluble pectin and sugar, the most important parts of TSS, seem to have a critical effect on the drink's rheological properties. Tables 7 and 8 show mean comparisons for apparent viscosity in all the produced drinks. Apparent viscosity increased by rising persimmon content and by adding gum. Apparent viscosities of 10% persimmon content samples with and without gum were 0.079 and 0.019 Pa.s, respectively. According to the reports from Homayouni Rad et al., (2012), these apparent viscosities were nearly like the viscosity of chocolate milk. They have reported that the viscosity of ordinary chocolate milk, chocolate milk without sugar, and chocolate milk with no sugar but with 6% inulin were 0.03513, 0.027, and 0.0352 Pa.s, respectively. Their results were similar to those of the present study (Homayouni Rad et al., 2012).

The obtained data from Herschel–Bulkley model and comparison of flow index and consistency coefficient means in the drinks with and without the gum are shown in Table 9. Consistency coefficient and yield stress increased by increasing persimmon content which had mainly sugar, and hydrocolloid substances like pectin. The drinks with 10% persimmon content with and without gum had the highest flow indexes of 0.90 and 0.96, respectively. They also had the lowest consistency coefficient 0.965 and 0.098 pa.s^n^, while the other drinks had lower and the same flow indexes. Data showed that by increasing persimmon content in all the drinks, flow indexes decreased (0.96 to 0.216). In all the drinks, consistency coefficients increased by increasing persimmon content. These changes are due to the dry solids (mainly sugar) and hydrocolloid substances in persimmon. Adding gum also increased consistency coefficients (Table 7). The effects of persimmon content and gum on flow index and consistency coefficient are depicted in Figure 4.

Homayouni Rad et al., (2012) and other researchers reported that by increasing inulin, viscosity and the quality of physical properties increased in chocolate milk (Bourne, 2002; Homayouni Rad et al., 2012). Researchers obtained the same results in producing a dairy drink by milk, a kind of jam (Cajeta), and ҡ‐carrageenan (Ramírez‐Sucre & Vélez‐Ruiz, 2011). They have reported that the drink without ҡ‐carrageenan did not have yield stress and its consistency coefficient was low (0.00369–0.00497 Pa.s^n^). Its flow index was 0.91–0.98, while consistency coefficient of the drink with 0.04%ҡ‐carrageenan was 0.071–0.153 Pa.s^n^, and yield stress was 0.131–0.329 Pa. Like the present study yield stress increased by increasing hydrocolloids in a dairy drink, other researchers also reported the same results (Abu‐Jdayil et al., 2004; Yanes et al., 2002).

Comparison of flow indexes and consistency coefficients in the drinks with and without gum using Student's *t*‐test revealed that 0.1% gum had a significant effect on the consistency coefficient in all the contents of persimmon and increased them (Table 8). Gum had a significant effect on flow indexes in both 10 and 20% persimmon contents, while 0.1% gum did not have any outstanding effect on the flow index in the drink with 30% persimmon content (*p* < .05).

## 5 CONCLUSION

By applying a proper content of persimmon in milk, we can produce a nutritious flavored milk drink with acceptable taste, stability, and consistency. Sugar, gum, and persimmon contents have remarkable effects on the drink acceptability, rheology, and consistency. Considering hydrocolloid substances in persimmon, producing a stable flavored milk drink without gum is suggested. The drinks with 20 and 30% amounts of persimmon are non‐Newtonian fluid with different yield stresses. The samples with 10% persimmon content with and without gum do not have yield stress, and are Newtonian. As this drink has high nutrient substances like phenol, dietary fiber, carotenoids, vitamins, antioxidants, etc., it can be more beneficial for human health.

## CONFLICT OF INTEREST

There is no conflict of interest in the present research.

## ETHICAL APPROVAL

This study does not involve any human or animal testing.

## INFORMED CONSENT

Written informed consent was obtained from all study participants.

## Data Availability

The data that support the findings of this study are available from the corresponding author upon reasonable request.
